# A refined technique for extraction of extracellular matrices from bacterial biofilms and its applicability

**DOI:** 10.1111/1751-7915.12155

**Published:** 2014-08-23

**Authors:** Akio Chiba, Shinya Sugimoto, Fumiya Sato, Seiji Hori, Yoshimitsu Mizunoe

**Affiliations:** 1 Department of Bacteriology, The Jikei University School of Medicine3–25-8, Nishi-Shimbashi, Minato-ku, Tokyo, 105-8461, Japan; 2 Department of Infectious Disease and Control, The Jikei University School of Medicine3–25-8, Nishi-Shimbashi, Minato-ku, Tokyo, 105-8461, Japan

## Abstract

Biofilm-forming bacteria embedded in polymeric extracellular matrices (ECMs) that consist of polysaccharides, proteins and/or extracellular DNAs (eDNAs) acquire high resistance to antimicrobial agents and host immune systems. To understand molecular mechanisms of biofilm formation and maintenance and to develop therapeutic countermeasures against chronic biofilm-associated infections, reliable methods to isolate ECMs are inevitable. In this study, we refined the ECM extraction method recently reported and evaluated its applicability. Using three *Staphylococcus aureus* biofilms in which proteins, polysaccharides or eDNAs are major contributors to their integrity, ECMs were extracted using salts and detergents. We found that extraction with 1.5 M sodium chloride (NaCl) could be optimum for not only ECM proteins but also polysaccharides and eDNAs. In addition, long-time incubation was not necessary for efficient ECM isolation. Lithium chloride (LiCl) was comparative to NaCl but is more expensive. In contrast to SDS, NaCl hardly caused leakage of intracellular proteins and did not affect viability of bacterial cells within biofilms. Furthermore, this method is applicable to other bacteria such as Gram-positive *Staphylococcus epidermidis* and Gram-negative *Escherichia coli* and *Pseudomonas aeruginosa*. Thus, this refined method is very simple, rapid, low cost and non-invasive and could be used for a broad range of applications.

## Introduction

Biofilms are intricate communities of microorganisms embedded in a self-produced matrix of extracellular polymer substances (EPS) and are adherent to an abiotic or biotic surface (Costerton *et al*., 1999). The extracellular matrix (ECM) consists of proteins (O'Neill *et al*., 2008), polysaccharides (O'Gara, 2007) and/or extracellular DNAs (eDNAs) (Mann *et al*., 2009). Extracellular matrix has diverse functions to maintain the structural integrity of the biofilm and to adapt to surrounding environments (Flemming and Wingender, 2010). A deleterious property of ECM for human beings is to confer resistance to antimicrobial agents (Davies, 2003) and host immune systems (Archer *et al*., 2011), which often becomes problematic in the clinical settings. Therefore, once biofilms are established on infected tissues or medical devices such as catheters and orthopaedic implants, it becomes difficult to eradicate them by chemotherapeutic strategies and biofilm-associated infections (e.g*.* catheter-related blood stream infections, prosthetic joint infections and artificial valve infections) become intractable and chronic (Del Pozo and Patel, 2009). In these cases, surgical intervention is often required to remove infected tissues or medical devices (Thwaites *et al*., 2011).

A large variety of bacteria exhibit the capacity to form biofilms in varying degrees (Dalton and March, 1998). The biofilm phenotypes differ between strains of a single species; for example, various strains of *Staphylococcus aureus* which is a major cause of biofilm-associated infections produce several types of biofilm with different ECM components. These biofilm phenotypes are simply classified according to their susceptibilities to ECM-degrading enzymes, because the formation and maintenance of biofilms definitively depend on the production and quality of ECMs. Conventionally, proteases (e.g*.* proteinase K), glycolytic enzymes (e.g*.* dispersin B) (Kaplan *et al*., 2004) and deoxyribonucleases (e.g*.* DNase I) are used for identifying proteinaceous, polysaccharide and DNA biofilms respectively (Sato *et al*., unpublished). If multiple components in an ECM are crucial for biofilm integrity, a combination of these enzymes, rather than single ones, is more effective in inhibiting the formation of biofilm and promoting its dispersal. In order to understand basic principles of the biofilm lifestyle at a molecular level, identification of ECM components is a primary important task. Although much effort to isolate ECM components have been reported (Liang *et al*., 1992; Tabouret *et al*., 1992; Tapia *et al*., 2009; Wu and Xi, 2009), efficient ECM isolation is still challenging in terms of cost performance, simplicity and/or applicability to various types of components and bacteria.

Recently, we reported a simple method for extracting ECMs from *S. aureus* biofilms using a high concentration of sodium chloride (NaCl) at 1 M (Sugimoto *et al*., 2013). A principle of this method may be similar to that of ion-exchange chromatography. Usually, bacterial surface are considered to be negatively charged due to the electrical state of outer components such as outer membranes (phospholipids and lipopolysaccharides) in Gram-negative bacteria and cell walls (peptidoglycans and teichoic acids) in Gram-positive ones. However, some positively charged molecules (membrane embedded proteins and cell wall-anchored proteins) are also displayed on bacterial surfaces. Therefore, there are negatively or positively charged loci on the surfaces. Extracellular matrix components (proteins, polysaccharides and eDNAs) are also thought to be positively or negatively charged depending upon individual characteristics under biofilm conditions used. Although Van der Waals force and hydrophobic interactions may not be ignored, ionic interactions seem to be an important driving force for the adherence of ECM constituents to bacterial surfaces (Dunne and Burd, 1992; Frølund *et al*., 1996; Jucker *et al*., 1996; Dunne, 2002). Higher ionic strength of Na^+^ and Cl^-^ ions might thus trigger the release of ECM components from bacterial cells (Fig. S1).

In this study, we refined our NaCl-based ECM extraction method using *S. aureus* biofilm models and compared with other methods previously reported elsewhere. We found that, using 1.5 M NaCl solution, ECM can be extracted from *S. aureus* cells more easily, inexpensively, rapidly and/or non-invasively compared with the other method using other chemicals such as sodium dodecyl sulfate (SDS) and lithium chloride (LiCl). Furthermore, our method was applicable to not only other Gram-positive *Staphylococcus epidermidis* but also Gram-negative *Escherichia coli* and *Pseudomonas aeruginosa*.

## Results and discussion

### Optimization of ECM extraction conditions

**Fig 1:**
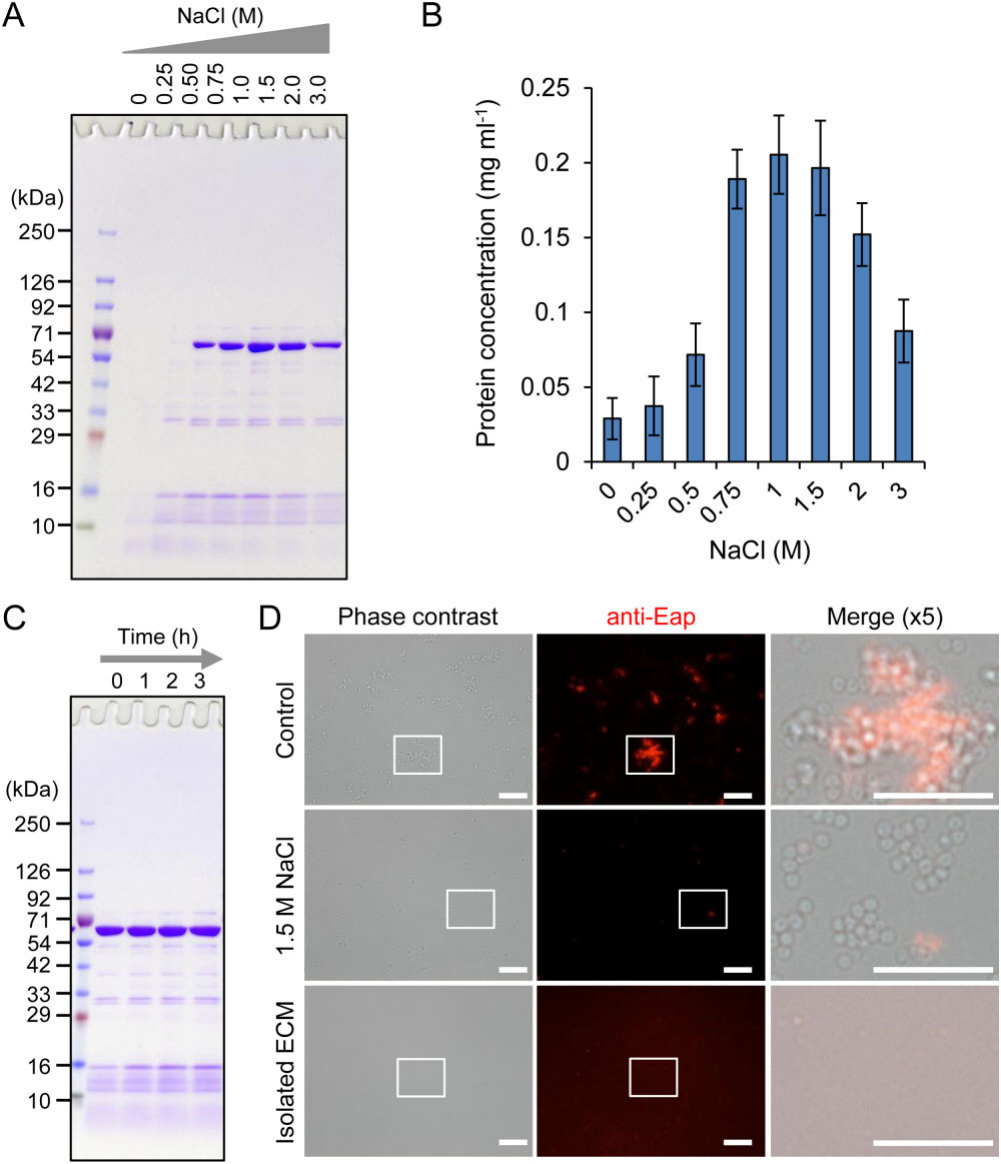
Sodium chloride is available for isolating ECM proteins from biofilms.A. Extracellular matrices of *S. aureus* MR23 were extracted with various concentrations of NaCl and were applied to SDS-PAGE with CBB staining.B. Protein concentrations in the extracted ECMs were quantified by Bradford method. The means and standard deviations of triplicate determinations are represented.C. Extracellular matrices were extracted after the indicated incubation periods in the presence of 1.5 M NaCl and were then dissolved by SDS-PAGE.D. MR23 biofilm cells treated with or without 1.5 M NaCl, and the extracted ECM were probed with anti-Eap primary and Cy3-labeled secondary antibodies and were observed with a fluorescence and phase-contrast microscope. Higher magnification (fivefold) images of the white rectangles in the phase contrast and fluorescence images are merged. Bars indicate 10 μm. The positions of molecular mass markers in kilodaltons (kDa) are shown at the left of each panel (A and C).

To optimize reagents for ECM extraction, we selected a biofilm model of clinically isolated methicillin-resistant *S. aureus* strain, MR23, since this strain produces a robust biofilm in which major components of the ECM are proteins including extracellular adherence protein (Eap) (Sugimoto *et al*., 2013). Of note, we used conical tube biofilms to ensure reproducibility and simplicity as shown in Fig. S1. In this method, planktonic and biofilm cells are incorporated into the pellet fraction at the first centrifugation step. If only biofilm cells are desired, planktonic cells can be removed by discarding the culture medium and gentle washing with certain buffers before the centrifugation. However, there is no significant difference in the profiles between the isolated ECM of biofilm cells and that of planktonic cells under the tested conditions (Fig. S2). We first extracted ECMs using various concentrations of NaCl and analysed them by SDS-PAGE (Fig. 1A). Several remarkable bands were detectable when ECMs were extracted with NaCl concentrations of 0.5 M and above. As previously reported (Sugimoto *et al*., 2013), the prominent band with molecular mass of 70 kDa corresponds to Eap that is a specific ECM protein of *S. aureus* (Hussain *et al*., 2008) and forms ECM architectures (Sugimoto *et al*., 2013). Therefore, we used Eap as an ECM marker of the MR23 biofilm. The band intensity of Eap increased in a dose-dependent manner, ranging from 0.5 to 1.5 M NaCl but decreased at 2 M and above. The result of protein quantification by Bradford method also showed a maximum peak at around 1–1.5 M NaCl (Fig. 1B). These results strongly suggest that 1.5 M is a proper concentration for ECM extraction. Henceforth, 1.5 M NaCl was used for further analyses. Next, we examined incubation time for the ECM isolation. There was no significant difference between the indicated incubation times at 25°C (Fig. 1C), revealing that the addition of 1.5 M NaCl could immediately lead to a detachment of ECMs from bacterial cells. Indirect immunofluorescence microscopy using anti-Eap polyclonal primary and Cy3-labeled secondary antibodies also demonstrated that Eap localized surface and extracellular milieu, and the signals disappeared almost completely after the 1.5 M NaCl treatment (Fig. 1D). Dispersed signals could be observed in the isolated ECM fraction probably due to the presence of solubilized Eap proteins.

**Fig 2:**
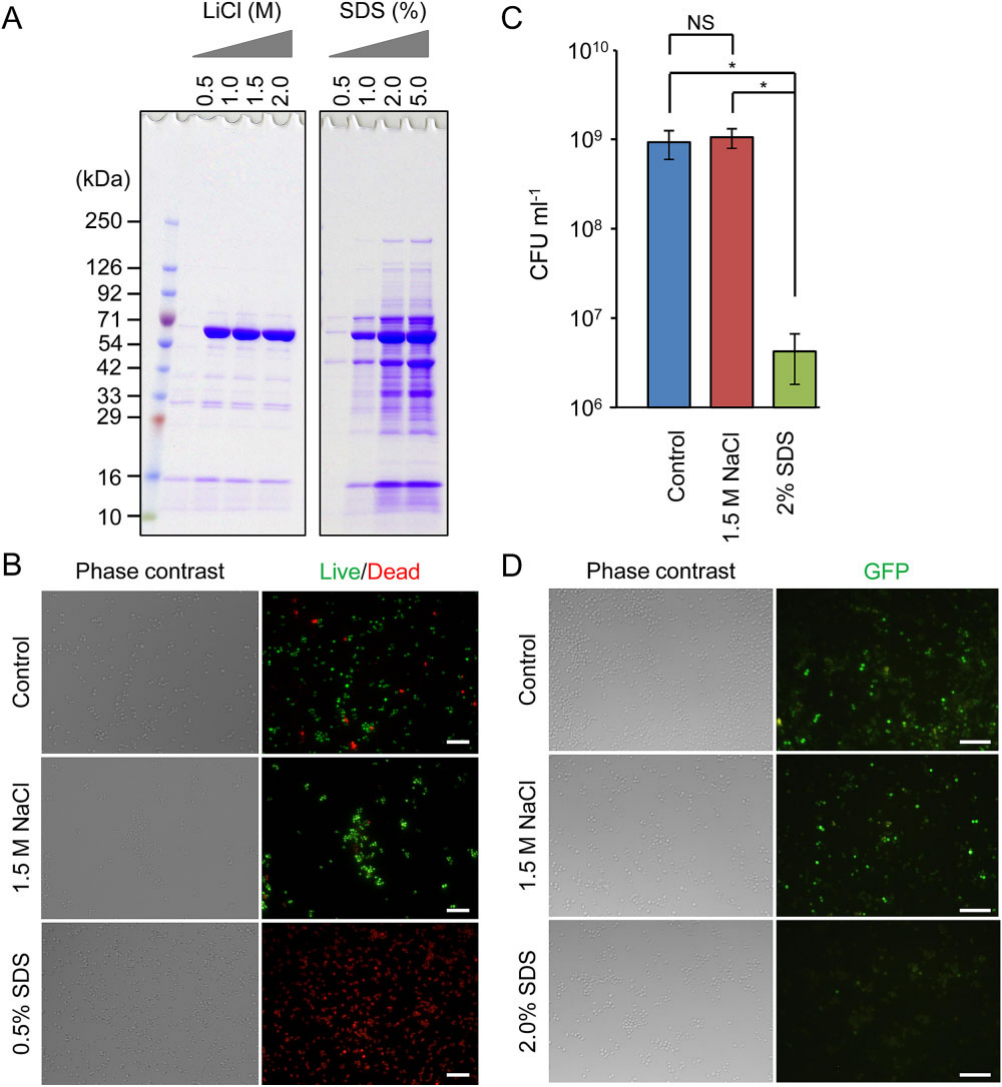
Assessment of cytotoxicity of NaCl and SDS.A. Extracellular matrices of *S. aureus* MR23 were extracted by the addition of the indicated concentrations of LiCl or SDS and were applied to SDS-PAGE with CBB staining. A molecular mass marker was also loaded to the left lane.B. Live/Dead staining images of MR23 biofilm cells after the treatment with 1.5 M NaCl or 0.5% (w/v) SDS are shown. A solution of 0.9% NaCl was used as a control. Phase contrast and fluorescence images are shown. Live and dead cells are stained in green and red respectively.C. Colony-forming unit of biofilm cells before and after the treatment with 1.5 M NaCl or 2% (w/v) SDS were measured. The means and standard deviations of triplicate determinations are represented.D. *Staphylococcus aureus* MR23 pP1GFP cells were treated with 1.5 M NaCl, 2% (w/v) SDS or PBS (as control) was observed with a fluorescence and phase-contrast microscope. Bars indicate 10 μm (B and D).

We next compared ECM extraction efficiency of NaCl with those of other salts [potassium chloride (KCl) and LiCl] and detergents (SDS, NP-40, Triton X-100 and Tween 20). The reasons why we selected these reagents are: (i) KCl is approximately equivalent to NaCl in terms of cost and conventional utility, (ii) LiCl was used for ECM extraction as reported previously (Liang *et al*., 1992) (iii) and some detergents, especially SDS, were generally used for protein solubilization (Tabouret *et al*., 1992). Potassium chloride was inconvenient, since the addition of high concentration of KCl into SDS sample buffer or running buffer led to precipitation of the ECM samples, making the analysis difficult (data not shown). In contrast, LiCl was found as efficient as NaCl in extracting ECMs as judged by SDS-PAGE (Fig. 2A), but LiCl is more expensive than NaCl. The detergents used in this study, except for SDS, were not meaningful for ECM extraction at all the concentrations tested (Fig. S3). On the other hand, the addition of SDS resulted in the recovery of a large variety of proteins including Eap (Fig. 2A). To address the issue that cytoplasmic and membrane proteins may leak to the extracellular milieu due to altered membrane permeability and integrity, we evaluated the effects of these chemicals on the viability of biofilm cells by live/dead staining and colony-forming units (CFU) counting. Notably, a large number of cells were alive but only a small population of cells were dead even in the case of non-treated cells, indicating that a minority of dead cells coexist with live cells in the biofilm as judged by live/dead staining (Fig. 2B). Although NaCl treatment did not affect the cell viability, SDS-treated cells were almost dead. Colony-forming unit counting support these result. Colony-forming unit of these SDS-treated cells was significantly reduced compared with those of control and NaCl-treated ones (*, *P* < 0.01) (Fig. 2C). In addition, fluorescence of cytoplasmic green fluorescent protein (GFP) in recombinant MR23 pP1GFP cells (Sugimoto *et al*., 2013) disappeared only when the cells were treated with SDS (Fig. 2D), suggesting that cytoplasmic GFP leaked out from the cells. Taken altogether, judging from efficiency, cost performance and non-invasiveness, NaCl is the best among the chemicals tested in this study.

The isolated ECMs will be used for further biochemical analyses to identify individual components and to examine their functional roles in biofilm development. If high concentrations of NaCl interfere with further analyses, they can be easily removed from the ECM fraction by the additional procedure such as dialysis and gel filtration chromatography. In our recent study, removal of NaCl was not necessary for identification of ECM proteins by a top-down proteome approach (Sugimoto *et al*., 2013). Although it is often difficult to purify a demanded ECM constituent apart from other components, by combining our ECM isolation method with a conventional chromatographic technique such as nickel-affinity column (Sugimoto *et al*., 2013) or fibrinogen-affinity column chromatography (Palma *et al*., 1999) (Fig. S4), we succeeded in purifying recombinant Eap protein and native one, supporting the merit of our method, too.

### Applicability of NaCl for extraction of ECMs containing polysaccharides and eDNAs

**Fig 3:**
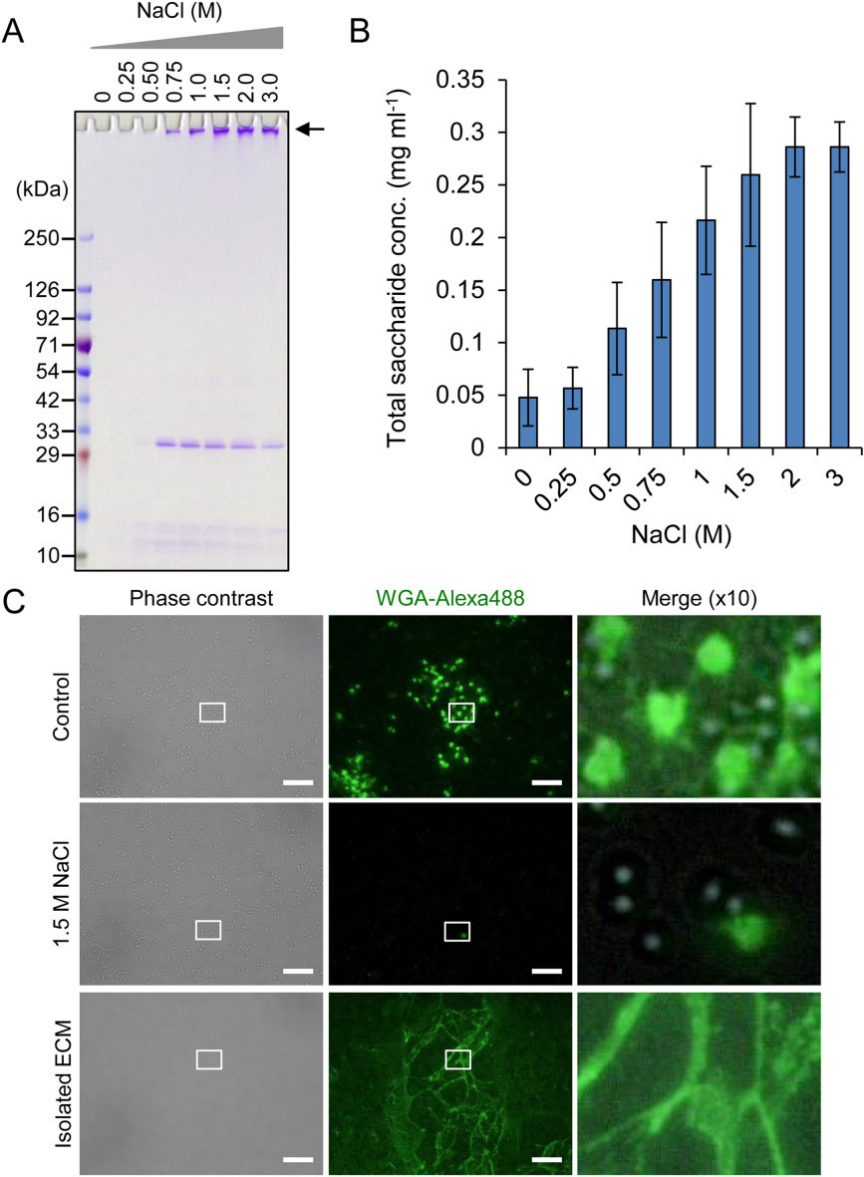
Sodium chloride is valuable in extracting ECM polysaccharides from biofilms.A. Extracellular matrices of *S. aureus* MR10 were extracted with various concentrations of NaCl and were dissolved by SDS-PAGE with CBB staining. The bands stacked at the top of the gel (shown as an arrow) are hallmark of polysaccharides as recently reported (Sugimoto *et al*., 2013). The positions of molecular mass markers in kilodaltons (kDa) are represented at the left.B. Total saccharide concentrations in the extracted ECM were quantified by phenol sulfuric acid method. The means and standard deviations of triplicate determinations are represented.C. MR10 biofilm cells with and without 1.5 M NaCl treatment and the extracted ECMs were stained with green fluorescence probe-labeled lectin (WGA-Alex488) and were observed with a fluorescence and phase-contrast microscope. Higher magnification (10-fold) images of the white rectangles in the phase contrast and fluorescence images are merged. Bars are 10 μm.

Here, we investigated the applicability of our newly refined method for extraction of polysaccharide components in ECMs. Polysaccharide intercellular adhesin (PIA), also known as poly N-acetylglucosamine (PNAG), is a major ECM constituent in certain *S. aureus* strains (Kaplan *et al*., 2004). The *S. aureus* MR10, clinically isolated in the Jikei hospital, produces a biofilm sensitive to dispersin B, but neither proteinase K nor DNase I (Sato *et al*., unpublished), suggesting that this strain produces a polysaccharide biofilm. We therefore used this strain in the present study and extracted ECMs from MR10 cells using various concentrations of NaCl. The profile of polysaccharides and proteins were analysed by SDS-PAGE (Fig. 3A). The band that is stacked at the top of the polyacrylamide gel and degraded by dispersin B is a feature of insoluble polysaccharides as recently reported (Sugimoto *et al*., 2013). The present study also showed that the stacked bands of the ECMs isolated from MR10 biofilms were degraded by dispersin B (Fig. S5). The polysaccharides bands were detected in the ECM samples extracted with NaCl concentrations of 0.75 M and above, and the intensity was not apparently changed at higher concentrations 1.5 M and above. The profile of total sugar concentration in the ECM determined by phenol sulfuric acid method (Fig. 3B) was parallel with the result of SDS-PAGE (Fig. 3A). Furthermore, the other bands with molecular mass of approximately 31 kDa were also detected in these ECM (Fig. 3A), and these bands were degraded by proteinase K but not dispersin B and DNase I (Fig. S5), denoting that the ECM of MR10 is composed of PIAs and protein(s). Judging from the result that the band intensity of the 31 kDa protein(s) was decreased at the NaCl concentrations of more than 1.5 M (Fig. 3A), 1.5 M NaCl is enough to extract polysaccharides and better to isolate polysaccharides and proteins simultaneously. Since diverse sugar monomers and linkages exist in bacterial extracellular polysaccharides (Flemming *et al*., 2007), carbohydrate chemical analyses are still challenging. Characterization and biochemical analysis of the polysaccharides in the ECM samples are of great interest and will be our future direction to understand molecular mechanisms of biofilm development.

To confirm whether polysaccharides were detached by NaCl treatment, they were stained with Alexa488-labeled wheat germ agglutinin (WGA-Alex488) (Fig. 3C). The fluorescent signals of polysaccharides were detected on surfaces and interspaces of the cells. Interestingly, networks of filamentous structures were also observed. After ECM extraction with 1.5 M NaCl, these signals disappeared almost completely. In addition, filamentous networks in the extracted ECM fraction bound to WGA-Alexa488. These results represent that 1.5 M NaCl is applicable to dispersal of ECM polysaccharides from *S. aureus* cells.

**Fig 4:**
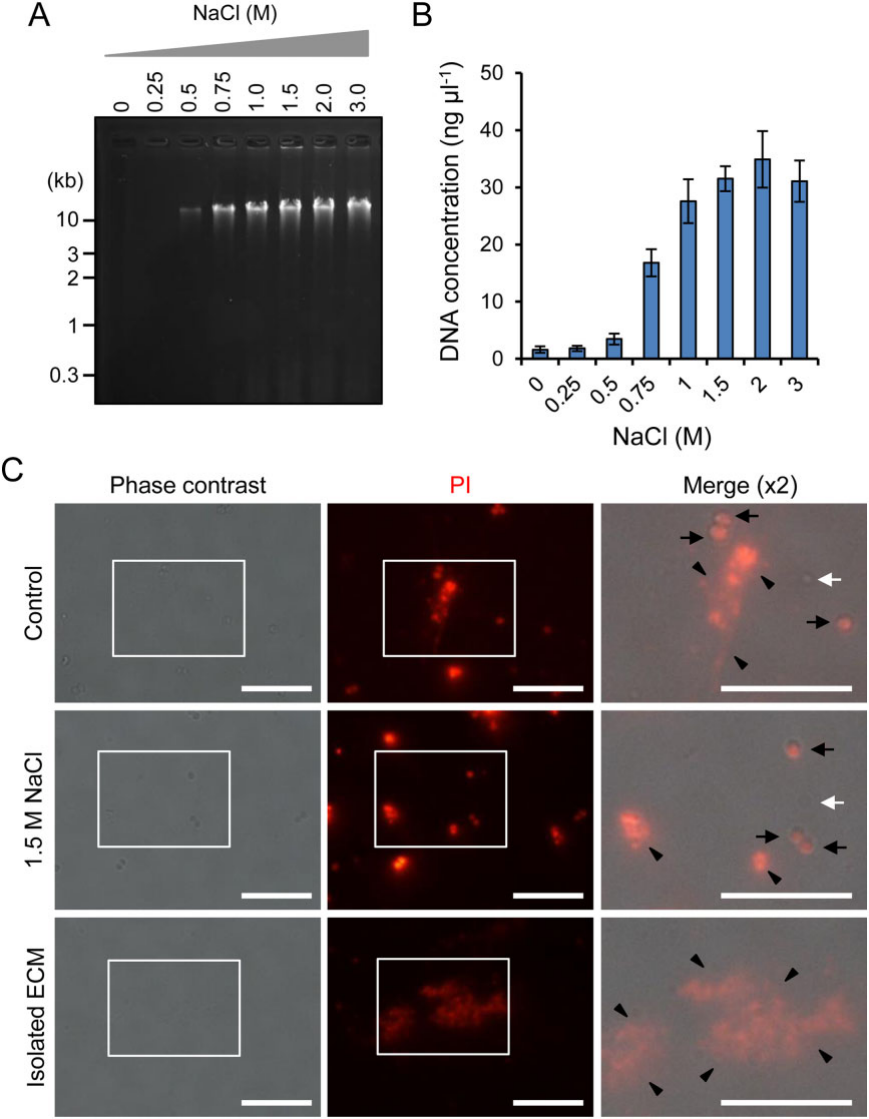
Extracellular DNAs are detached from biofilms by the addition of NaCl and harvested quantitatively.A. Extracellular matrices from *S. aureus* MS10 biofilms were extracted with the indicated concentrations of NaCl and were subjected to agarose gel electrophoresis. The gel was stained with ethidium bromide. The positions of molecular mass markers in kilobase pairs (kb) are shown at the left.B. Extracellular DNAs in the extracted ECM were purified to remove contaminated proteins and quantified by measuring absorbance at 260 nm. The means and standard deviations of triplicate determinations are represented.C. MS10 biofilm cells treated with or without 1.5 M NaCl and the extracted ECM were stained with PI and were observed with a fluorescence and phase-contrast microscope. Higher magnification (twofold) images of the white rectangles in the phase contrast and fluorescence images are merged. Black arrows, white arrows and black arrowheads represent bacterial cells stained with PI (dead cells), non-stained cells (variable cells) and eDNAs respectively. Bars indicate 10 μm.

To clarify whether NaCl can be used for extraction of ECMs composed of not only proteins and polysaccharides but also eDNAs, we selected a biofilm model of *S. aureus* MS10 strain which produces a DNase I-sensitive biofilm (Sato *et al*., unpublished). Extracellular matrices of MS10 biofilms were also extracted by the addition of various concentrations of NaCl and were analysed by agarose gel electrophoresis. In the 1.5% agarose gel, prominent bands with a huge molecular mass were detected in the ECM samples extracted with NaCl at 0.5 M and above (Fig. 4A). In addition, eDNAs were also detected in the ECM factions of MR23 and MR10 (Fig. S6). According to the migration of DNAs in the agarose gel, they seem to be whole genomic DNAs released from the cells probably due to autolysis. Since the isolated ECMs of MS10 contained a small proportion of proteins as in the case of those from MR10 (Fig. S5), we purified DNA in the extracted ECMs using a DNA purification kit and determined the concentration of DNA by measuring the absorbance at 260 nm. The yield of DNA increased with the dose of NaCl and reached a plateau at 1.5 M or 2 M (Fig. 4B). This profile was apparently equivalent to the data obtained by agarose gel electrophoresis (Fig. 4A). Furthermore, we stained eDNAs with propidium iodide (PI) and observed them under fluorescence microscopy. In addition to intracellular DNAs within dead cells, PI-positive signals of eDNAs were observed in the control sample, but the signals of eDNAs became invisible almost completely after the extraction of ECM with 1.5 M NaCl. In addition, clusters of DNA were observed in the extracted ECM fraction (Fig. 4C).

These results together indicate that 1.5 M NaCl could be used for isolation of diverse types of ECMs from *S. aureus* biofilms.

### Applicability of NaCl for extraction of ECMs from other bacteria

**Fig 5:**
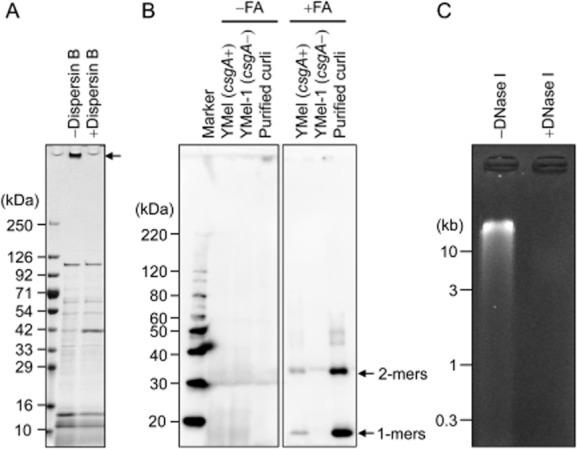
Applicability of the ECM extraction method to *S. epidermidis*, *E. coli* and *P. aeruginosa* biofilms.A. Extracellular matrices extracted from *S. epidermidis* SE04 by the addition of 1.5 M NaCl were treated with or without dispersin B and were then applied to SDS-APGE. The gel was stained with CBB. An arrow indicates polysaccharides.B. Extracellular matrices of *E. coli* YMel and its isogenic *csgA* mutant YMel-1 were isolated with 1.5 M NaCl. Curli amyloid fibres in the ECM fraction were treated with or without formic acid. Purified curli was also used as a positive control. The proteins were analysed by Western blotting using anti-CsgA antibody. Arrows indicate monomeric and dimeric CsgA. FA, formic acid.C. Extracellular matrices of *P. aeruginosa* PAO1 were extracted with 1.5 M NaCl and were subjected to agarose gel electrophoresis. The extracted ECMs were treated with or without DNase I. The gel was stained with ethidium bromide. The positions of molecular mass markers in kilodaltons (kDa) (A and B) and kilobase pairs (kb) (C) are shown at the left of each panel respectively.

Finally, we tested whether the ECM-isolation method sophisticated in this study is applicable to other Gram-positive and Gram-negative bacteria. Gram-positive bacterium *S. epidermidis* is also a major cause of biofilm diseases in clinical settings, and one of the major components of its ECM is PIA whose biosynthesis is regulated by *ica* gene products (O'Gara, 2007). Using the same procedure with *S. aureus*, ECM was isolated from *S. epidermidis* SE04, a clinically isolated strain that forms a PIA-positive biofilm (Sato *et al*., unpublished). As in the case of MR10, the bands stacked at the top of the polyacrylamide gel were detected, and they were completely degraded by dispersin B (Fig. 5A). In addition, proteinaceous components were also detected in the gel, indicating the presence of certain proteins that may contribute to the formation and maintenance of *S. epidermidis* biofilms.

Previously, isolation of TasA amyloid fibres from *Bacillus subtilis* biofilms by using 1 M NaCl has been reported (Romero *et al*., 2010), suggesting that high concentrations of NaCl is useful for extracting not only natively folded or denatured proteins but also ordered protein aggregates. Here, we use an *E. coli* biofilm model in which curli amyloid fibres are major components of the ECM and prominently contribute to the structural integrity of the biofilm (Maeyama *et al*., 2004). The curli-dependent biofilm that formed when a wild-type YMel strain was grown in YESCA plates at 25°C for 3 days was collected and used for ECM extraction. Here, we selected CsgA, a major structural component of curli, as a maker of the ECM in *E. coli*. The ECM extracted with 1.5 M NaCl was treated with formic acid to depolymerize curli amyloid fibres into CsgA monomers (Chapman *et al*., 2002) and Western blotting using an anti-CsgA antiserum (Fig. 5B). In parallel, curli was purified by conventional homogenization and centrifugation procedures (Chapman *et al*., 2002) and was used as a positive control. The ECM extracted from the isogenic *csgA* mutant of YMel (YMel-1) was also used as a negative control (Kikuchi *et al*., 2005). We detected specific bands corresponding to CsgA monomers (17 kDa band) in the ECM fraction of YMel (Fig. 5B).

In *P. aeruginosa,* eDNA is an integral structural component in biofilms (Whitchurch *et al*., 2002). We tested whether eDNA could be extracted from colony biofilms of *P. aeruginosa* PAO1 strain. As shown in Fig. 5C, eDNA in the isolated ECM was detected by agarose gel electrophoresis, and the signal disappeared when treated with DNase I.

It is well known that *staphylococci* have salinity tolerance. We wondered whether 1.5 M NaCl may cause cell death of *E. coli* and *P. aeruginosa* cells. Therefore, we assessed cell damages caused by 1.5 M NaCl in these Gram-negative bacteria. As shown in Fig. S7, CFUs of these bacteria were not significantly reduced after the treatment with 1.5 M NaCl compared with those of PBS, suggesting that the ECM extraction method does not cause severe damages leading to cell death. Taken altogether, these findings support the notion that high concentrations of NaCl are applicable to isolation of ECMs from various microorganisms including not only Gram-positive bacteria but also Gram-negative ones.

### Conclusion

We refined ECM extraction method for various bacterial biofilms. The merits of this method are its simplicity and applicability. This method may be useful for typing of biofilms produced by diverse bacteria. Typing of biofilms at the strain level is also an important issue, since biofilm phenotypes differ between strains. Typing of biofilms has conventionally been conducted by testing their susceptibility to ECM-degrading enzymes. Our results imply that biofilms are not composed of only single component, even though they are susceptible to an ECM-degrading enzyme. Characterization of the individual ECM constituents rather than susceptibility to ECM-degrading enzymes may be more important for understanding how biofilms are developed and maintained and for taxonomical and epidemiological investigations.

## Experimental procedures

### Bacterial strains and culture media

Bacterial strains used in this study were listed in Table S1 in the supplemental material. *Staphylococcus aureus* and *S. epidermidis* were grown at 37°C in brain heart infusion (BHI) medium (Becton Dickinson, Franklin Lakes, NJ, USA) or BHI medium supplemented with 1% (w/v) glucose (BHIG). *Escherichia coli* strains were grown in Luria-Bertani (LB) medium (Merk, Darmstadt, Germany) or YESCA plate composed of 1% (w/v) casamino acid (Becton Dickinson), 0.1% (w/v) yeast extract (Becton Dickinson) and 2% (w/v) agar plate. *Pseudomonas aeruginosa* was cultured in LB medium and LB plate containing 2% agar.

To reduce non-specific immunoglobulin G (IgG) binding in immunofluorescence microscopy, the *srtA* gene, which encodes sortase A (Mazmanian *et al*., 2001), was deleted by in-frame deletion using pKOR1 (Bae and Schneewind, 2006). Because sortase A cleaves the LPXTG motif of cell wall-anchoring proteins including protein A, which is one of the major IgG binding proteins in *S. aureus* (Forsgren and Sjöquist, 1966)*.* Briefly, approximately 500 bp upstream and downstream sequences of the *srtA* gene were amplified by PCR from MR23 genomic DNA using the following primer sets respectively: *attB1*-*srtA*-F (5'-ggggacaagtttgtacaaaaaagcaggctttaataatcttattttcactcgttatctta-3’) and *srtA*-R (5'-attcatccattagcgtaatagaacgttaaggctccttttataca-3’); *srtA*-F (5'- tgtataaaaggagccttaacgttctattacgctaatggatgaat-3’) and *attB2-srtA*-R (5'- ggggaccactttgtacaagaaagctgggttaaccatctattaaatttaaaacctacatt-3’). These fragments were connected by splicing by overlap extension polymerase chain reaction (Horton *et al*., 1989). The created PCR product was cloned into pKOR1 by using Gateway BP Clonase II enzyme mix (Life Technologies, Palo Alto, CA, USA), and the resulting plasmid was named as pKOR1-*ΔsrtA* (Table S1). Using pKOR1-*ΔsrtA*, the *srtA* gene was deleted from the MR23 genomic DNA according to the procedure reported previously (Bae and Schneewind, 2006).

### Reagents for ECM extraction

Sodium chloride was purchased from Wako (Osaka, Japan). Lithium chloride and SDS were purchased from Nacalai Tesque (Kyoto, Japan). All reagents were dissolved into distilled deionized water to the indicated concentrations, and the resulting solutions were supplemented with a protease inhibitor cocktail (Nacalai) to block degradation of proteins in the isolated ECM.

### ECM-degrading enzymes

Dispersin B from *Aggregatibacter actinomycetemcomitans* (20 μg ml^−1^, Kane Biotech Inc., Manitoba, Canada), Proteinase K from *Tritirachium album* (100 μg ml^−1^, Sigma, St Louis, MO, USA) and DNase I (100 U ml^−1^, Roche Diagnostics, Mannheim, Germany) were used for dispersal of preformed biofilms or degradation of ECM components.

### Antibodies and fluorescent dyes

An anti-Eap polyclonal antibody was raised in a rabbit against purified Eap (Sugimoto *et al*., 2013) (Scrum, Tokyo, Japan). The anti-Eap IgG was purified with a MAbTrap Kit (GE Healthcare, Buckinghamshire, UK) according to the manufacturer's instructions. A rabbit anti-CsgA polyclonal antibody was raised against the CsgA peptide (LDQWNGKNSEMTVKQFGGGN) (Loferer *et al*., 1997) with the C-terminal carrier cysteine residue (Medical and Biological Laboratories, Aichi, Japan). Horseradish peroxidase (HRP)-conjugated goat anti-rabbit IgG and Cy3-labeled goat anti-rabbit IgG were purchased from Bio-Rad Laboratories (Hercules, CA, USA) and GE Healthcare respectively. WGA-Alexa488, SYTO9 and PI were purchased from Life Technologies.

### Biofilm formation and ECM extraction

Staphylococcal biofilms were grown as follows: single colonies grown on BHI plates were inoculated into 3 ml of BHI medium and incubated overnight at 37°C with shaking. The overnight cultures were 1000-fold diluted in 10 ml of BHIG medium in a conical tube (15 ml, Becton Dickinson) and were incubated at 37°C for 24 h under static conditions. After the incubation, the conical tubes were centrifuged at 8000 g for 10 min at 25°C, and the supernatants were discarded completely. To extract ECM components, the residual pellets were suspended with the indicated regents (100 μl) at various concentrations. The suspensions were centrifuged at 5000 g for 10 min at 25°C, and the supernatants were transferred to a new test tube (1.5 ml) as ECM fractions (Fig. S1B). If required, the suspensions were incubated for the indicated periods at 25°C before centrifugation.

*Escherichia coli* and *P. aeruginosa* colony biofilms were cultivated on YESCA agar plates at 25°C for 72 h and on LB agar plates at 37°C for 24 h respectively. After the incubation, the colony biofilms were scraped with a scraper and suspended with 1 ml of 1.5 M NaCl solution. These suspensions were centrifuged at 5000 g for 10 min at 25°C without any incubation period. The supernatants were harvested as ECM fractions.

### Electrophoreses for proteins, polysaccharides and eDNAs in the isolated ECM fractions

To analyse protein and polysaccharide components in the extracted ECM fractions, they were subjected to SDS-PAGE. Here, 5% to 20% (w/v) polyacrylamide gradient gels (Atto, Tokyo, Japan) were used to separate proteins with a broad range of molecular masses and polysaccharides which are stacked on the top of the gels. Five microlitres of ECM samples were applied per lane. After the electrophoresis, the gels were stained with Coomassie Brilliant Blue (CBB).

For DNA analysis, extracted ECMs were separated by 1.5% (w/v) agarose gel electrophoresis. Ten microlitres of the fractions were applied per lane. After the electrophoresis, the gels were stained with ethidium bromide according to a conventional procedure.

### Western blotting

To assess whether ECM components of *E. coli* biofilms can be isolated by the addition of 1.5 M NaCl, CsgA, a major structural component of curli amyloid fibres, was detected by Western blotting, since YMel, an *E. coli* K-12 strain, produces a curli-dependent biofilm under the tested conditions (Maeyama *et al*., 2004). The ECM fractions from YMel and its isogenic *csgA* mutant, YMel-1, were separated by SDS-PAGE as mentioned above. As a control, curli purified from YMel biofilms was also used as a positive control. If required, curli amyloid fibres were depolymerized with 90% formic acid (Sigma) as previously reported (Chapman *et al*., 2002). The proteins were separated by SDS-PAGE and transferred to a polyvinylidene difluoride membrane. The membrane was treated with blocking solution composed of 5% (w/v) skim milk and Tris buffered saline containing 0.1% Tween 20 (TBS-T) overnight at 25°C. After gentle washing with TBS-T, the membrane was probed with an anti-CsgA primary antibody (1000-fold diluted in TBS-T) for 1 h at 25°C. The membrane was washed three times with TBS-T and was subsequently incubated with the secondary antibody (200 000-fold diluted HRP-conjugated goat anti-rabbit IgG in TBS-T) for 1 h at 25°C. After three times washing with TBS-T, the protein-related signals were detected using ECL prime (GE healthcare) on a LAS 4000 luminescent image analyser (GE healthcare).

### Quantification of proteins

We quantified proteins in the isolated ECM fractions using a Bradford protein assay kit (Bio-Rad) according to manufacturer's protocol. The concentration was measured at 590 nm with Infinite F200 Pro (Tecan, Männedorf, Switzerland). Bovine serum albumin (BSA) was used as a standard.

### Quantification of total saccharide

Total saccharide concentrations in the ECMs were measured by phenol sulfuric acid method. Twenty microlitres of the isolated ECM was mixed with 20 μl of 5% phenol in the 96 wells plate. Then, 100 μl of sulfuric acid was added and incubated for 10 min at 25°C. The concentration was measured at 492 nm with Infinite F200 Pro (Tecan). Glucose was used as a standard.

### Quantification of DNA

To quantify DNA in the isolated ECM fractions, we purified the DNA using a Promega SV Wizard DNA purification system (Promega, Madison, WI, USA) according to manufacturer's protocol. The concentration of the purified DNA was measured with NanoDrop 2000 (Thermo Fisher Scientific, Waltham, MA, USA).

### Fluorescence microscopy

To observe the distribution of Eap, a major ECM component in *S. aureus* MR23, immunofluorescence microscopy was performed using anti-Eap primary and Cy3-labeled secondary antibodies. Small aliquots (10 μl) of non-treated MR23 biofilm cells, 1.5 M NaCl-treated ones and the extracted ECM were spotted on a slide glass and fixed with 10% (w/v) paraformaldehyde for 10 min at 25°C. One hundred microlitres of blocking solution [PBS containing 0.5% (w/v) BSA and 5% (v/v) goat serum] was placed on the slide and removed after overnight at 25°C. The primary antibody (100-fold diluted anti-Eap antibody in the blocking solution, 100 μl) was placed on the slide and left for 1 h at 25°C. The sample on the slide was then washed three times with PBS. Remaining solution was removed with paper pieces. Then, in a dark room, the secondary antibody (200-fold diluted Cy3-labeled goat anti-rabbit IgG in the blocking solution, 100 μl) was placed on the slide and removed after 1 h by washing five times with PBS. The immunostained sample was covered with one drop of a mounting medium (Vector Laboratories, Burlingame, CA, USA) and a cover glass. The immunostained cells on the slide were observed with a fluorescence and phase-contrast microscope.

To visualize ECM polysaccharides, biofilm cells of MR10 were stained with WGA-Alexa488. Aliquots (10 μl) of the non-treated biofilm cells, 1.5 M NaCl-treated ones, and the extracted ECM were placed on a slide glass and air-dried for 30 min at 25°C. After twice washing with PBS, the cells were covered with WGA-Alexa488 (5 μg ml^−1^) and removed after 20 min at 37°C. The cells were washed two times with PBS and covered with one drop of the mounting medium and a cover glass. The lectin-stained samples on the slide were observed with a fluorescence and phase-contrast microscope.

To detect eDNAs, the biofilm cells of MS10 were stained by PI (Life Technologies). One hundred microlitres of non-treated biofilm cells, 1.5 M NaCl-treated ones and the extracted ECM were mixed with 0.3 μl of PI solution in the test tube and incubated for 15 min at 25°C in the dark room. Aliquots of the solution (5 μl) were placed on a slide glass and covered with a cover glass. The stained samples on the slide were observed with a fluorescence and phase-contrast microscope.

### Cytotoxicity assay

Viability of MR23 biofilm cells were evaluated by counting CFU after the treatment with 1.5 M NaCl, 2% (w/v) SDS or 0.9% (w/v) NaCl (control). To distinguish dead cells from live ones, they were stained with PI and SYTO9 (Life Technologies), respectively, according to manufacturer instruction and were observed with a fluorescence and phase-contrast microscope. The leakage of cytoplasmic proteins was confirmed by observing fluorescence of GFP as a marker of cytoplasmic protein. Here, we used a recently constructed recombinant *S. aureus* strain, MR23 pP1GFP (Table S1) (Sugimoto *et al*., 2013). The biofilm cells were treated with 1.5 M NaCl, 2.0% SDS or PBS (control) and were subsequently observed under a fluorescence and phase-contrast microscope.

## Supporting Information

**Fig. S1.** The principle of the method employed in extracting bacterial extracellular matrices (ECMs) composed of proteins, polysaccharides and/or eDNAs. A. Graphical image of bacterial cells embedded in ECMs. ECM components including proteins, polysaccharides and eDNAs can bind to bacterial cells or each other via electrostatic attractive forces, ionic attractive forces, hydrophobic interactions and van der Waals interactions (Flemming and Wingender, 2010). Increasing concentrations of salts and detergents (e.g*.* NaCl and SDS) potentially disrupt these interactions by coating cell surfaces and ECM constituents and therefore lead to detachment of ECMs from the cells. This image is an example using 1.5 M NaCl. B. The procedure for preparation of biofilm and isolation of ECMs. Biofilms are grown in an appropriate medium (e.g*.* BHIG: brain heart infusion medium containing 1% glucose) in a conical tube. The bacterial cells covered with a self-produced ECM are sedimented by centrifugation at 8000 g for 10 min at 25°C. The cells and the ECM are suspended in an appropriate solution (e.g*.* 1.5 M NaCl). The suspension is immediately centrifuged at 5000 g for 10 min at 25°C, and the ECM components in the supernatant detached from bacterial cells are transferred to a new tube. **Fig. S2.** SDS-PAGE profiles of planktonic and biofilm cells. Biofilms are grown in a conical tube as described in Fig. S1. Before the first centrifugation, cultured medium in which many planktonic cells existed was harvested and transferred to another conical tube. The residual bacterial cells attached to the tube were used as biofilm cells. Extracellular matrices extracted from planktonic and biofilm cells with 1.5 M NaCl and were analysed by SDS-PAGE. There is no significant difference in the profile of proteins between them. **Fig. S3.** Non-ionic detergents are not effective in detachment of ECMs from biofilm cells. Using the indicated percentages (w/v) of Triton X100, Tween 20 and Nonidet 40 (NP-40), ECM proteins of *S. aureus* MR23 were extracted according to the procedure shown in Fig. S1 and were subjected to SDS-PAGE. The gel was stained with CBB. The positions of molecular mass markers in kDa are shown at the left. **Fig. S4.** Purification of Eap from the isolated ECM of *S. aureus* MR23. A. Native Eap was purified form the isolated ECM of *S. aureus* MR23 with a fibrinogen-affinity column. The isolated ECM (input), flow through, wash and elution fractions with PBS or acetic acid were subjected to SDS-PAGE. B. The concentrated fractions of flow through, wash and elution were also analysed by SDS-PAGE. The wash fraction contained highly purified Eap, and in addition, the elution fraction contained both Eap and Efb with some contaminated proteins. The positions of molecular mass markers in kDa are shown at the left (A and B). **Fig. S5.** Susceptibilities of ECMs to Proteinase K, DNase I and Dispersin B. ECMs were extracted from *S. aureus* MR10 and MS10 biofilms and were treated with Proteinase K (100 μg ml^−1^), DNase I (100 U ml^−1^) and Dispersin B (20 μg ml^−1^) for 2 h at 37°C. They were subsequently applied to SDS-PAGE with CBB staining. The positions of molecular mass markers in kDa are shown at the left. Proteins in the ECMs from MR10 and MS10 were degraded by Proteinase K. In the case of the ECM from MR10, bands stacked at the top of the gel disappeared after the treatment with Dispersin B, and thus they are judged as polysaccharides as recently reported (Sugimoto *et al*., 2013). Interestingly, their profiles are significantly different from each other and from that of MR23, suggesting the applicability of this method to typing of biofilms. **Fig. S6.** Analyses of eDNAs in the ECMs isolated from *S. aureus* MR23, MR10 and MS10. The ECMs isolated from *S. aureus* MR23, MR10 and MS10 biofilms and treated with the indicated enzymes as described in Fig. S4 and applied to agarose gel electrophoresis. Non-treated ECMs were also used as a control. The gels were stained with ethidium bromide. The positions of molecular mass markers in kb are shown at the left. Notably, the bands were sensitive to DNase I neither Proteinase K nor Dispersin B, indicating that they contains DNA molecules to varying degrees. **Fig. S7.** Assessment of cell damages for *E. coli* and *P. aeruginosa.* To assess cell damages for *E. coli* and *P. aeruginosa* caused by extraction with NaCl, CFUs of biofilm cells were counted. Briefly, colony biofilms on the plate were scraped and suspended with PBS. Then, this suspension was separated into two tubes equally. These samples were centrifuged at 8000 g for 10 min at 25°C. After centrifugation, the supernatants were discarded. One of these pellets was suspended with 1.5 M NaCl solution, and the other one was suspended with PBS (as control). These suspensions were centrifuged at 5000 g for 10 min at 25°C. After centrifugation, the supernatants were discarded. The pellets were suspended with PBS and the CFU was counted. A. Colony-forming unit of *E. coli* biofilm cells before and after the treatment with PBS and 1.5 M NaCl for YMel strain. The means and standard deviations of triplicate determinations are represented. B. Colony-forming unit of *P. aeruginosa* biofilm cells before and after the treatment with PBS and 1.5 M NaCl for PAO1 strain. The means and standard deviations of triplicate determinations are represented.

**Table S1.** Bacterial strains and plasmids used in this study.

## References

[b1] Archer NK, Mazaitis MJ, Costerton JW, Leid JG, Powers ME, Shirtliff ME (2011). *Staphylococcus aureus* biofilms: properties, regulation, and roles in human disease. Virulence.

[b2] Bae T, Schneewind O (2006). Allelic replacement in *Staphylococcus aureus* with inducible counter-selection. Plasmid.

[b4] Chapman MR, Robinson LS, Pinkner JS, Roth R, Heuser J, Hammar M (2002). Role of *Escherichia coli* curli operons in directing amyloid fiber formation. Science.

[b5] Costerton JW, Stewart PS, Greenberg EP (1999). Bacterial biofilms: a common cause of persistent infections. Science.

[b6] Dalton HM, March PE (1998). Molecular genetics of bacterial attachment and biofouling. Curr Opin Biotechnol.

[b7] Davies D (2003). Understanding biofilm resistance to antibacterial agents. Nat Rev Drug Discov.

[b8] Del Pozo JL, Patel R (2009). Clinical practice. Infection associated with prosthetic joints. N Engl J Med.

[b9] Dunne WM (2002). Bacterial adhesion: seen any good biofilms lately?. Clin Microbiol Rev.

[b10] Dunne WM, Burd EM (1992). The effects of magnesium, calcium, EDTA, and pH on the *in vitro* adhesion of *Staphylococcus epidermidis* to plastic. Microbiol Immunol.

[b11] Flemming HC, Wingender J (2010). The biofilm matrix. Nat Rev Microbiol.

[b12] Flemming HC, Neu TR, Wozniak DJ (2007). The EPS matrix: the ‘house of biofilm cells. J Bacteriol.

[b13] Forsgren A, Sjöquist J (1966). ‘Protein A’ from *S. aureus*: I. Pseudo-immune reaction with human γ-globulin. J Immunol.

[b14] Frølund B, Palmgren R, Keiding K, Nielsen PH (1996). Extraction of extracellular polymers from activated sludge using a cation exchange resin. Water Res.

[b15] Horton RM, Hunt HD, Ho SN, Pullen JK, Pease LR (1989). Engineering hybrid genes without the use of restriction enzymes: gene splicing by overlap extension. Gene.

[b16] Hussain M, von Eiff C, Sinha B, Joost I, Herrmann M, Peters G, Becker K (2008). *eap* gene as novel target for specific identification of *Staphylococcus aureus*. J Clin Microbiol.

[b17] Jucker BA, Harms H, Zehnder AJ (1996). Adhesion of the positively charged bacterium *Stenotrophomonas**Xanthomonas**maltophilia* 70401 to glass and Teflon. J Bacteriol.

[b18] Kaplan JB, Velliyagounder K, Ragunath C, Rohde H, Mack D, Knobloch JK, Ramasubbu N (2004). Genes involved in the synthesis and degradation of matrix polysaccharide in *Actinobacillus actinomycetemcomitans* and *Actinobacillus pleuropneumoniae* biofilms. J Bacteriol.

[b19] Kikuchi T, Mizunoe Y, Takade A, Naito S, Yoshida S (2005). Curli fibers are required for development of biofilm architecture in *Escherichia coli* K-12 and enhance bacterial adherence to human uroepithelial cells. Microbiol Immunol.

[b20] Liang OD, Ascencio F, Fransson LA, Wadström T (1992). Binding of heparan sulfate to *Staphylococcus aureus*. Infect Immun.

[b21] Loferer H, Hammar M, Normark S (1997). Availability of the fibre subunit CsgA and the nucleator protein CsgB during assembly of fibronectin-binding curli is limited by the intracellular concentration of the novel lipoprotein CsgG. Mol Microbiol.

[b22] Maeyama R, Mizunoe Y, Anderson JM, Tanaka M, Matsuda T (2004). Confocal imaging of biofilm formation process using fluoroprobed *Escherichia coli* and fluoro-stained exopolysaccharide. J Biomed Mater Res A.

[b23] Mann EE, Rice KC, Boles BR, Endres JL, Ranjit D, Chandramohan L (2009). Modulation of eDNA release and degradation affects *Staphylococcus aureus* biofilm maturation. PLoS ONE.

[b24] Mazmanian SK, Ton-That H, Schneewind O (2001). Sortase-catalysed anchoring of surface proteins to the cell wall of *Staphylococcus aureus*. Mol Microbiol.

[b25] O'Gara JP (2007). *ica* and beyond: biofilm mechanisms and regulation in *Staphylococcus epidermidis* and *Staphylococcus aureus*. FEMS Microbiol Lett.

[b26] O'Neill E, Pozzi C, Houston P, Humphreys H, Robinson DA, Loughman A (2008). A novel *Staphylococcus aureus* biofilm phenotype mediated by the fibronectin-binding proteins, FnBPA and FnBPB. J Bacteriol.

[b27] Palma M, Haggar A, Flock JI (1999). Adherence of *Staphylococcus aureus* is enhanced by an endogenous secreted protein with broad binding activity. J Bacteriol.

[b3] Romero D, Aguilar C, Losick R, Kolter R (2010). Amyloid fibers provide structural integrity to *Bacillus subtilis* biofilms. Proc Natl Acad Sci USA.

[b28] Sugimoto S, Iwamoto T, Takada K, Okuda K, Tajima A, Iwase T, Mizunoe Y (2013). *Staphylococcus epidermidis* Esp degrades specific proteins associated with *Staphylococcus aureus* biofilm formation and host-pathogen interaction. J Bacteriol.

[b29] Tabouret M, de Rycke J, Dubray G (1992). Analysis of surface proteins of Listeria in relation to species, serovar and pathogenicity. J Gen Microbiol.

[b30] Tapia JM, Muñoz JA, González F, Blázquez ML, Malki M, Ballester A (2009). Extraction of extracellular polymeric substances from the acidophilic bacterium *Acidiphilium 3.2Sup(5)*. Water Sci Technol.

[b31] Thwaites GE, Edgeworth JD, Gkrania-Klotsas E, Kirby A, Tilley R, Török ME (2011). Clinical management of *Staphylococcus aureus* bacteraemia. Lancet Infect Dis.

[b32] Whitchurch CB, Tolker-Nielsen T, Ragas PC, Mattick JS (2002). Extracellular DNA required for bacterial biofilm formation. Science.

[b33] Wu J, Xi C (2009). Evaluation of different methods for extracting extracellular DNA from the biofilm matrix. Appl Environ Microbiol.

