# Supplementary material for: A refined technique for extraction of extracellular matrices from bacterial biofilms and its applicability
Source: Microb Biotechnol. 2014 Aug 23;8(3):392–403. doi: 10.1111/1751-7915.12155 (PMC4408173; doi:10.1111/1751-7915.12155)

Chiba and Sugimoto *et al*. Figure S1

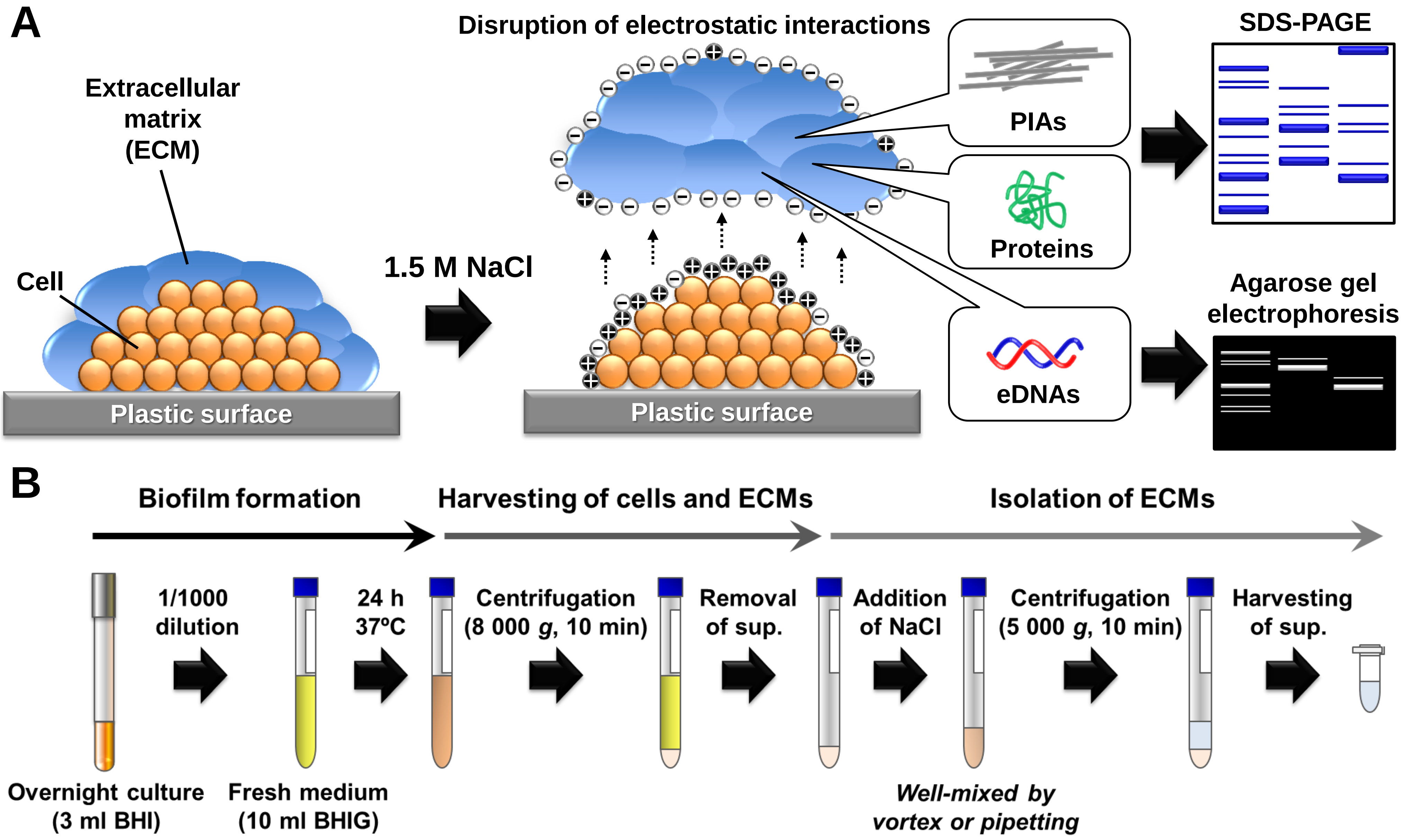

Chiba and Sugimoto *et al*. Figure S2

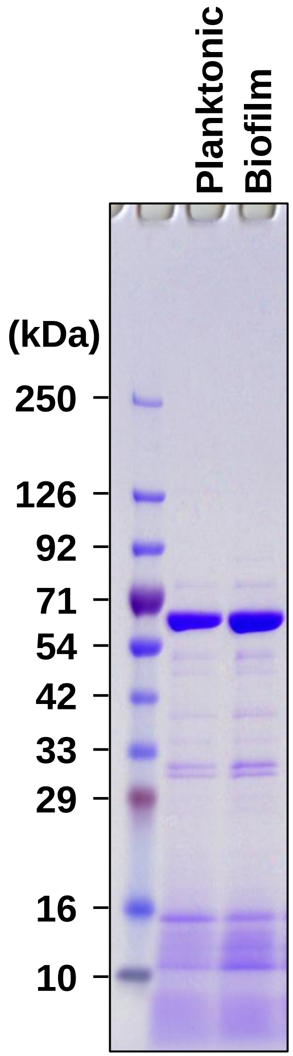

Chiba and Sugimoto *et al*. Figure S3

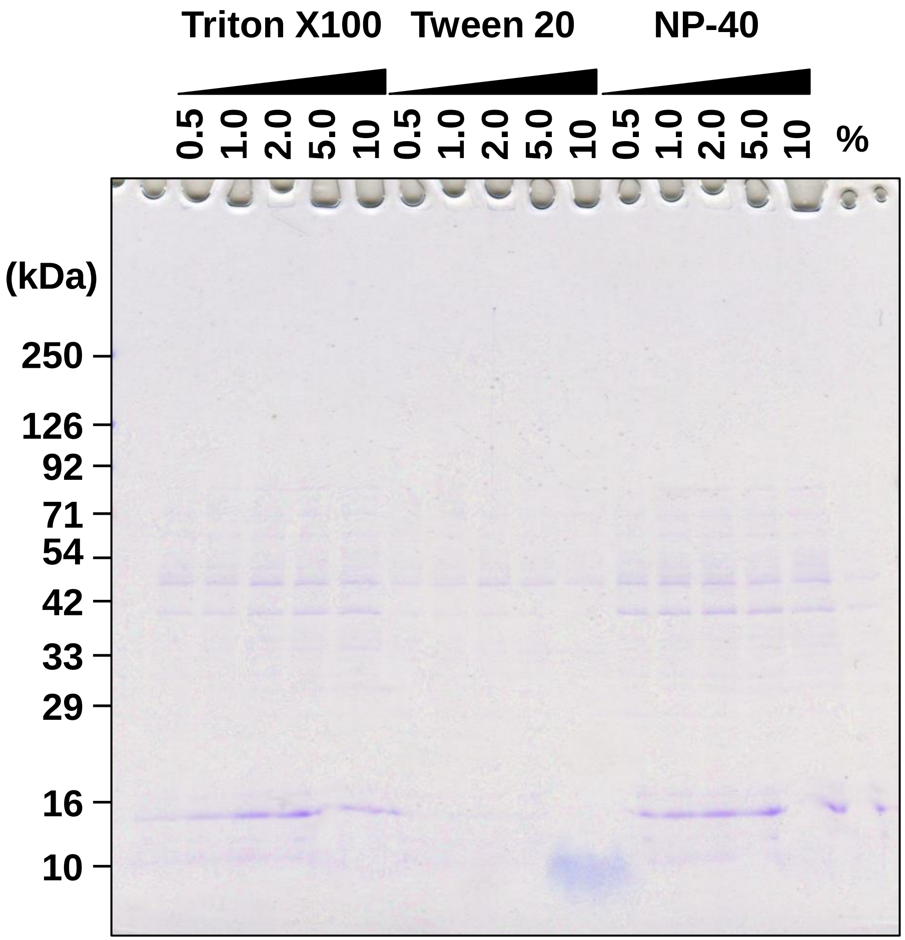

Chiba and Sugimoto *et al*. Figure S4

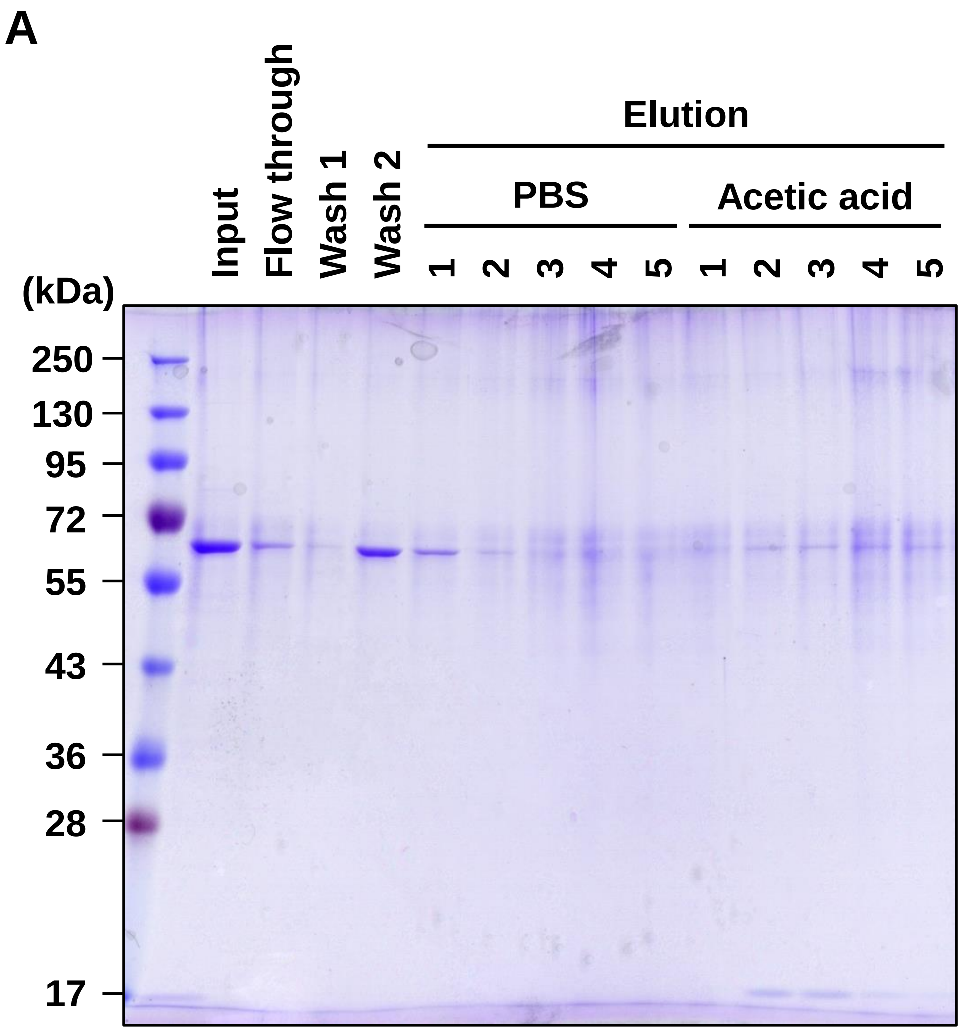

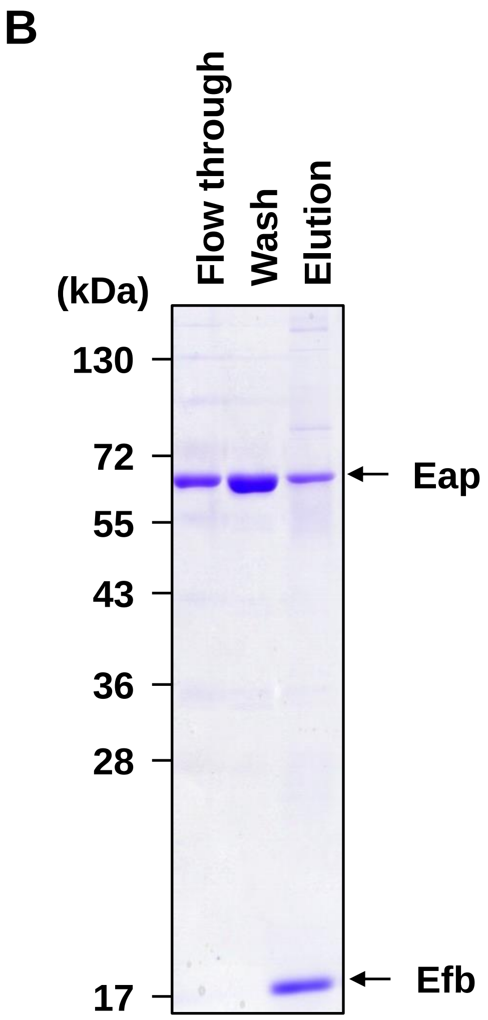

Chiba and Sugimoto *et al*. Figure S5

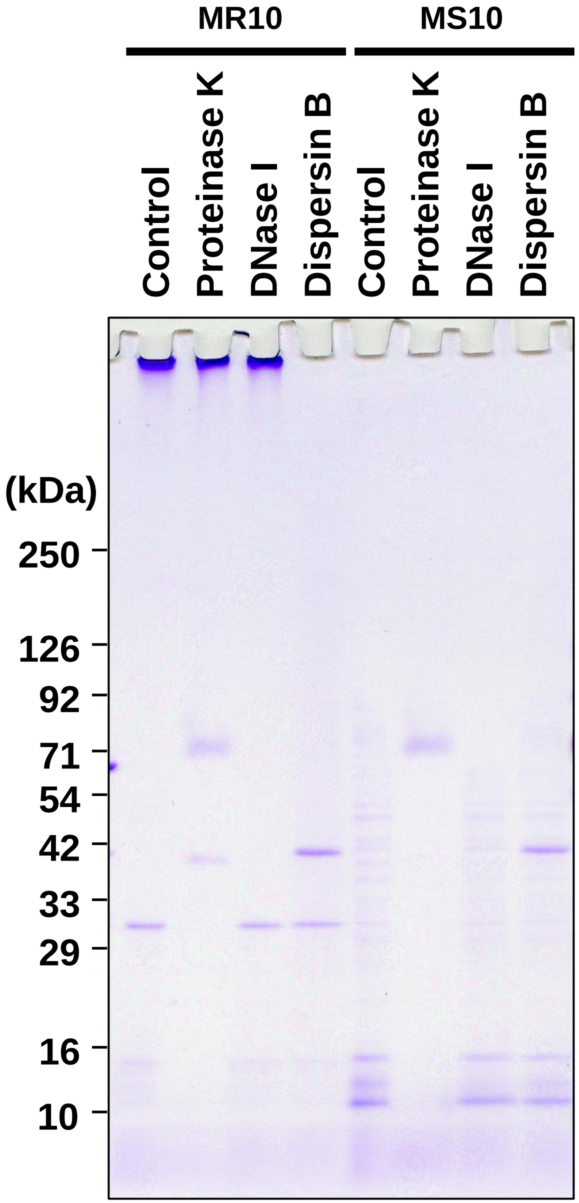

Chiba and Sugimoto *et al*. Figure S6

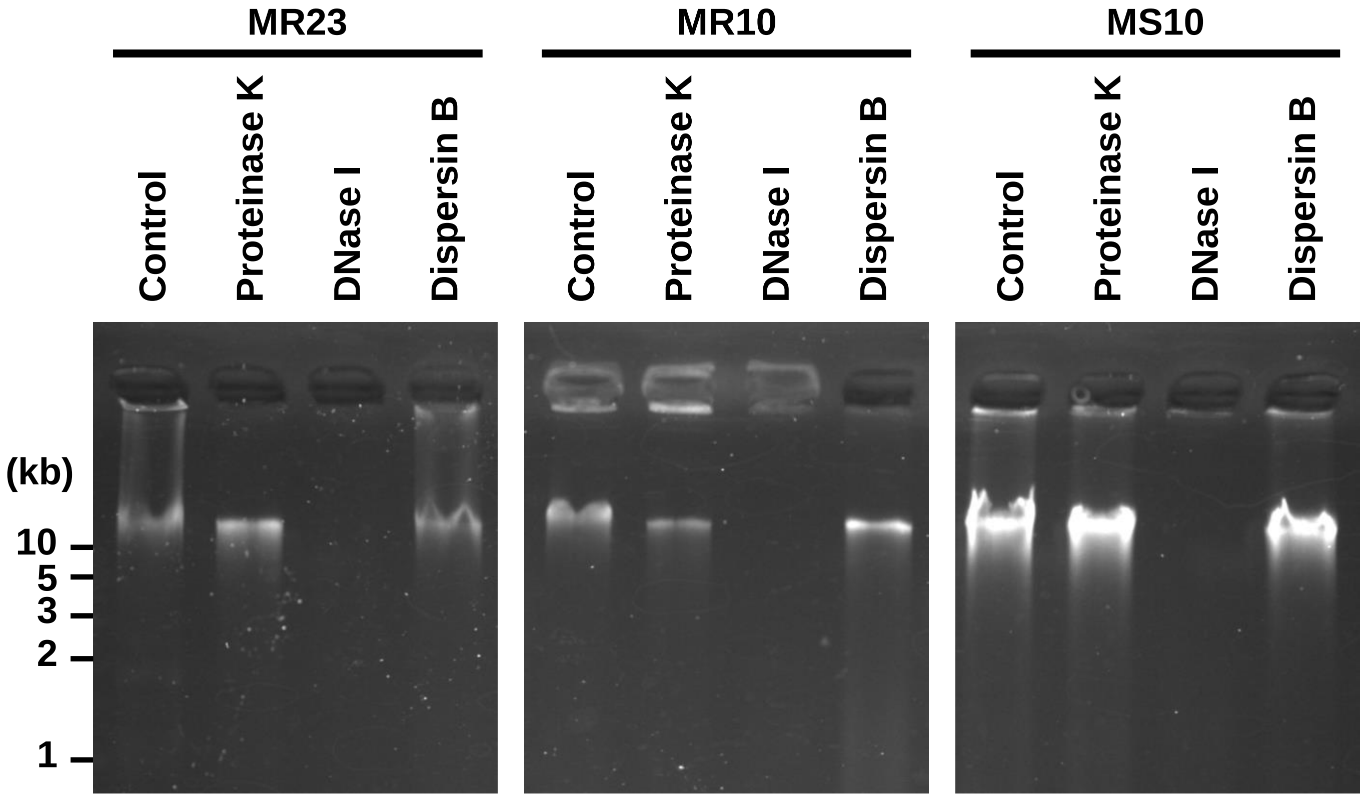

Chiba and Sugimoto *et al*. Figure S7

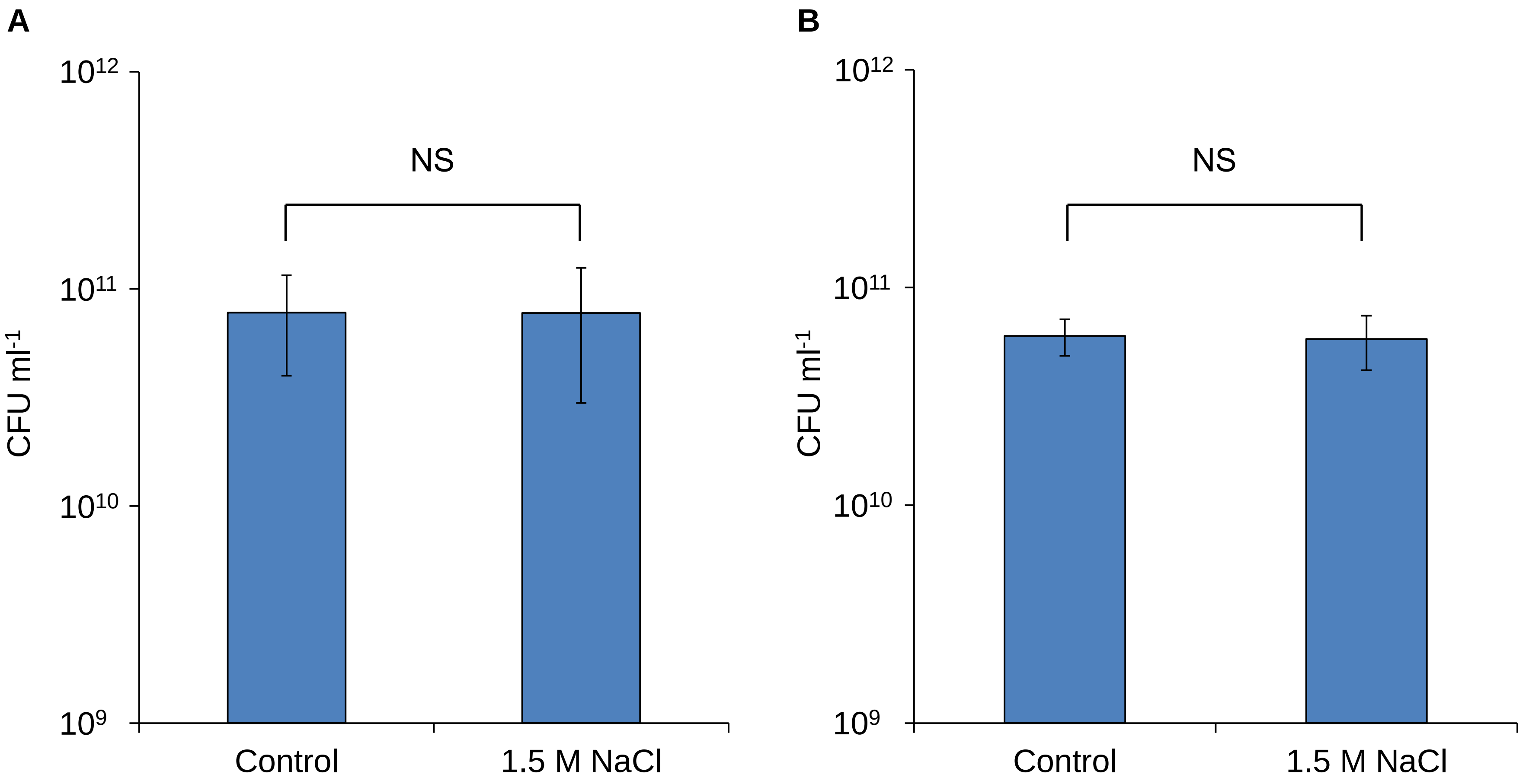

Supplement: Supplementary file 1 [file mbt20008-0392-sd1.pdf]
