# Supplementary material for: A refined technique for extraction of extracellular matrices from bacterial biofilms and its applicability
Source: Microb Biotechnol. 2014 Aug 23;8(3):392–403. doi: 10.1111/1751-7915.12155 (PMC4408173; doi:10.1111/1751-7915.12155)
Supplement: Supplementary file 2 [file mbt20008-0392-sd2.pdf]

Chiba and Sugimoto *et al*. Table S1

Table S1. Bacterial strains and plasmids used in this study.

Strains and plasmids	Description[1]	Source and reference
S. aureus strains		
RN4220	Restriction deficient derivative of 8325-4 for using sub-cloning host.	Kreiswirth *et al*., 1983
MR10	A clinical isolate of MRSA from the Jikei hospital, the biofilm was destructed by Dispersin B	Sato *et al*., unpublished
MS10	A clinical isolate of MSSA from the Jikei hospital, the biofilm was destructed by DNase I	Sato *et al*., unpublished
MR23	A clinical isolate of MRSA from the Jikei hospital, the biofilm was destructed by Proteinase K	Sugimoto *et al*., 2013
MR23 pP1GFP	MR23 transformed with pP1GFP, Nm^R	Sugimoto *et al*., 2013
MR23 Δ*srtA*	The *srtA* gene was deleted from MR23	This study
S. epidermidis strains		
SE04	Clinical isolate from the Jikei hospital, the biofilm was destructed by Dispersin B	Sato *et al*., unpublished
P. aeruginosa strains		
PAO1	The biofilm of this strain was contained eDNA.	Whitchurch *et al*., 2002
E. coli strains		
DH5α	*fhuA2 Δ(argF-lacZ)U169 phoA glnV44 Φ80 Δ(lacZ)M15 gyrA96 recA1 relA1 endA1 thi-1 hsdR17*	Toyobo (Osaka, Japan)
YMel	A derivative of K-12 strain, high level production of curli	Maeyama *et al*., 2004
YMel-1	The *csgA* gene was deleted from YMel	Maeyama *et al*., 2004
Plasmid		
pP1GFP	A plasmid for intracellular expression of GFP_{uv}, Nm^R	Sugimoto *et al*., 2013
pKOR1	An *E. coli/S. aureus* shuttle vector plasmid for knock out of staphylooccal genes by allelic exchange, Cm^R, Amp^R	Bae and Schneewind, 2006
pKOR1-Δ*srtA*	A pKOR1-derivative plasmid for knock out of the MR23 *srtA* gene, Cm^R, Amp^R	This study

[1] Amp^R: Ampicillin resistance; Cm^R: Chloramphenicol resistance; Nm^R: Neomycin resistance.
